# Long-lived Aqueous Rechargeable Lithium Batteries Using Mesoporous LiTi_2_(PO_4_)_3_@C Anode

**DOI:** 10.1038/srep17452

**Published:** 2015-12-09

**Authors:** Dan Sun, Yougen Tang, Kejian He, Yu Ren, Suqin Liu, Haiyan Wang

**Affiliations:** 1 College of Chemistry and Chemical Engineering, Central South University, Changsha, 410083, P.R. China; 2 Advanced Research Centre, Central South University, Changsha, 410083, P.R. China; 3 Battery Materials, Basf China Limited, Shanghai, 201206, P.R. China; 4 State Key Laboratory for Powder Metallurgy, Central South University, Changsha 410083, P.R. China

## Abstract

The instability of anode materials during cycling has been greatly limiting the lifetime of aqueous rechargeable lithium batteries (ARLBs). Here, to tackle this issue, mesoporous LiTi_2_(PO_4_)_3_@C composites with a pore size of 4 nm and a large BET surface area of 165 m^2^ g^−1^ have been synthesized by a novel two-step approach. The ARLB with this type of LiTi_2_(PO_4_)_3_@C anode, commercial LiMn_2_O_4_ cathode and 2 M Li_2_(SO_4_) aqueous solution (oxygen was removed) exhibited superior cycling stability (a capacity retention of 88.9% after 1200 cycles at 150 mA g^−1^ and 82.7% over 5500 cycles at 750 mA g^−1^) and excellent rate capability (discharge capacities of 121, 110, 90, and 80 mAh g^−1^ based on the mass of LiTi_2_(PO_4_)_3_ at 30, 150, 1500, and 3000 mA g^−1^, respectively). As verified, the mesoporous structure, large surface area and high-quality carbon coating layer of the LiTi_2_(PO_4_)_3_@C composite contribute to the breakthrough in achieving excellent electrochemical properties for ARLB.

Lithium ion batteries (LIBs) have dominated the portable electronic markets and also attracted overwhelming attentions for large-scale energy storage system (ESS) and electric vehicles (EVs)^1^, but the issues such as high cost and safety hazards arising from the usage of flammable organic electrolytes greatly limit its broader applications. As a result, new energy storage systems with low cost and high reliability are urgently needed^2^. Aqueous rechargeable lithium batteries (ARLBs), which use inexpensive salt solution as electrolyte, could fundamentally settle the safety issue and also avoid rigorous assembly conditions^3^,^4^. Furthermore, higher ionic conductivity of electrolyte and more environmental benignness could be achieved for ARLBs compared with non-aqueous LIBs^3^,^4^.

Unfortunately, its poor cycling stability is still a big challenge for ARLBs due to more complicate lithium intercalation processes in aqueous electrolyte^5^. The choice of available electrode materials, in particular, the anode materials, are largely limited because of the narrow stable window of water. Accordingly, the commercial cathode materials in LIBs including LiFePO_4_^6^, LiNi_1/3_Co_1/3_Mn_1/3_O_2_^7^, LiCoO_2_^8^,^9^,^10^, and LiMn_2_O_4_^11^ have been well studied as the cathodes for ARLBs. The anode for ARLBs requires the electrode materials with a Li^+^ intercalation potential of 2~3 V *vs.* Li^+^/Li^12^. There are only several kinds of suitable candidates, *e.g.,* vanadates and LiTi_2_(PO_4_)_3_. The first ARLB of VO_2_//LiMn_2_O_4_ reported by Dahn *et al.*^13^ can just cycle for 25 cycles. Since then, the ARLB systems such as LiV_3_O_8_//LiMn_2_O_4_, LiV_3_O_8_//LiNi_0.81_Co_0.19_O_2_, NaV_3_O_8_//LiMn_2_O_4_, NaV_6_O_15_//LiMn_2_O_4_ and new aqueous battry systems have been constructed^2^,^14^,^15^,^16^,^17^,^18^,^19^. Many of these systems, however, only displayed limited cycling stability due to the vanadium dissolution in aqueous solution and degradation of crystal structure, especially at a low current density^5^. LiTi_2_(PO_4_)_3_/C has shown the potential as anode for ARLB with relatively high power density and good cycling stability. By eliminating the soluble oxygen in Li_2_SO_4_ solution, LiTi_2_(PO_4_)_3_//LiFePO_4_ ARLB constructed by Xia *et al.*^20^ demonstrated a 1000 cycle life at a high current rate of 6C. However, the cycling stability of such ARLB system at low rates was still insufficient (85% after 50 cycles at a current rate of 8 hrs for a full charge/discharge test). Hence, a breakthrough in cycling life, particularly at a lower current density is urgently required for further applications of ARLBs.

There has been a consensus that LiMn_2_O_4_ and LiFePO_4_ could be used as advanced cathodes for ARLBs. As reported by Wu *et al.*^21^, porous LiMn_2_O_4_ nanograins could be stably cycled up to 10000 cycles with a capacity retention of 93% at a rate of 9C. In contrast, the instability of anode mainly caused by H_2_O attacking, dissolution of active materials and repetitive volume expansion has been remaining as a key issue for ARLBs. Pristine LiTi_2_(PO_4_)_3_ often shows a low electronic conductivity^22^, which could be greatly improved by reducing the particle size to nanoscale thanks to their shortened electron/Li ions diffusion paths^23^. Meanwhile, coating strategy with high quality carbon layer could not only significantly enhance the conductivity of materials but also protect active material from electrolyte corrosion, resulting in better cycling stability^24^. It is clear that the characteristics of carbon layer (*e.g.,* content, thickness, uniformity and structure), which can significantly affect the coating quality, generally depend on the selected carbon sources and the coating methods^25^,^26^. In our previous work, LiTi_2_(PO_4_)_3_ with high-quality carbon coating has been obtained and it demonstrated excellent cycling life with a capacity retention of 90% after 300 cycles at 0.2C^27^.

In the present work, LiTi_2_(PO_4_)_3_@C composite with mesoporous structure and homogeneous carbon coating was successfully fabricated by a solvothermal process combined with an annealing treatment. It is suggested that mesoporous composite could not only well accommodate the volume change during cycling by abundant pores but also provide reduced lengths for both mass and charge transports thanks to this unique structure^23^. More importantly, high quality carbon coating was achieved by using phenolic resin as carbon source and an *in situ* coating strategy. The as-prepared LiTi_2_(PO_4_)_3_@C composite exhibits superior electrochemical properties. The strategies proposed in this paper are absolutely template free and very facile for practical application.

## Results

**Figure 1:**
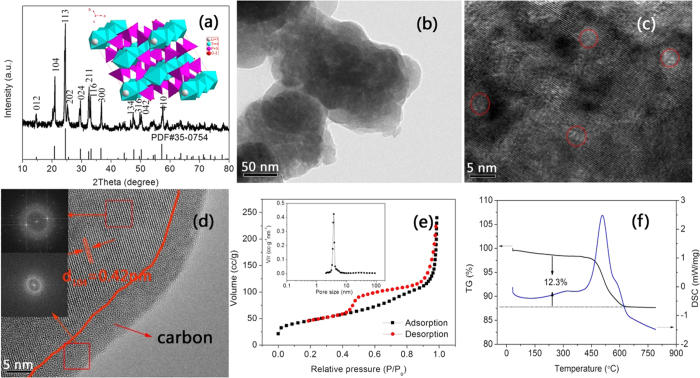
(**a**) XRD pattern of as-prepared LiTi_2_(PO_4_)_3_@C composite and crystal structure of LiTi_2_(PO_4_)_3_ (inset), (**b**) TEM image, (**c,d**) HRTEM images (the insets are the FFT images of corresponding red square), (**e**) N_2_ adsorption-desorption isotherm and Barrett-Joyner-Halenda (BJH) pore size distribution plot (inset), (**f**) Differential scanning calorimetry/thermal gravimetry (DSC/TG) of as-prepared LiTi_2_(PO_4_)_3_@C composite.

The X-ray diffraction (XRD) pattern (a) and transmission electron microscopy (TEM) image (b) of the precursor obtained by solvothermal process are shown in Figure S1, in which a very low crystallinity and an average particle size of 5 nm can be seen. The XRD pattern of as-prepared material after being sintered with carbon source is displayed in Fig. 1a. The diffraction peaks can be well indexed into LiTi_2_(PO_4_)_3_ phase with a rhombohedral NASICON type structure and a *R*3*c* space group (JCPDS#35–0754). The measured lattice parameters *a* = 0.8464 nm and c = 2.1442 nm are in good agreement with the previous reports^28^,^29^. The calculated average crystal size of LiTi_2_(PO_4_)_3_ from XRD pattern based on the Debye-Scherrer equation is 32.7 nm. The high resolution X-ray photoelectron spectroscopy (XPS) spectrum of Ti (Figure S2) confirms the existence of Ti^4+^ in LiTi_2_(PO_4_)_3_@C^30^. Figure 1a (inset) shows the crystal structure of LiTi_2_(PO_4_)_3_. The three-dimensional (3D) anionic framework is formed by corner-sharing PO_4_ tetrahedra and TiO_6_ octahedra, leaving large interconnected channels which can be occupied by Li ions^31^. This rigid 3D crystal structure could be a promising host for Li^+^ insertion/extraction. The TEM image in Fig. 1b indicates that LiTi_2_(PO_4_)_3_@C is composed of individual particles with a size range of 30–50 nm, which is close to the primary crystalline grain size (32.7 nm) obtained from Debye-Scherrer equation. Note that slight agglomeration takes place. The high resolution TEM (HRTEM) image (Fig. 1c) of the composite reveals clear lattice fringes with many cavities (as marked by red oval), indicating the existence of mesopores. The appearance of mesopores may originate from the assembly and recrystallization process of ultra-fine precursor nanoparticles (5 nm, Figure S1) and the decomposition of phenolic resin into carbon^32^. The study of detailed formation mechanism is still under way. The N_2_ adsorption-desorption isotherm of as-prepared LiTi_2_(PO_4_)_3_@C is shown in Fig. 1e, from which it can be seen that the LiTi_2_(PO_4_)_3_@C shows a typical IV isotherm and has a large Brunauer-Emmett-Teller (BET) surface area of 165 m^2^ g^−1^. The large surface area may be due to its mesoporous morphology (inset in Fig. 1e) and the carbon coating. A narrow size distribution of 4 nm is observed, in good agreement with the HRTEM result (Fig. 1c). It should be noted that high surface area and mesoporous structure can significantly improve the electrode/electrolyte contact, facilitate the Li ions transport, and enhance the utilization efficiency of the material. More importantly, it is the mesoporous structure that enables is able to accommodate strain/stress during the Li ion insertion/extraction process^21^.

**Figure 2:**
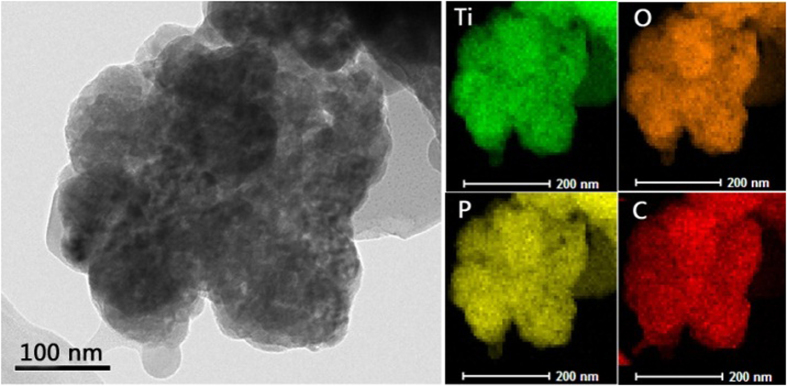
STEM-EDS elemental mapping images of as-prepared mesoporous LiTi_2_(PO_4_)_3_@C composite.

The uniformity and structure features of carbon coating were also investigated. As seen from Fig. 1d, a uniform carbon layer with a thickness of *ca.* 5 nm can be observed on the edge of particle. The Fast Fourier Transform Algorithm (FFT) image further confirms its amorphous nature. The carbon coating with a thickness of 4–8 nm on electrode materials could reach a good balance of *e*^*−*^ conductivity and Li^+^ diffusion, promising superior electrochemical properties. The regular lattice fringe and its corresponding FFT image validate the crystal nature of as-prepared LiTi_2_(PO_4_)_3_. The inter-planar spacing deduced from the Fig. 1d is 0.42 nm, agreeing well with the d-spacing of the (104) plane of rhombohedral LiTi_2_(PO_4_)_3_. The carbon content of LiTi_2_(PO_4_)_3_@C is measured to be 12.3wt% by DSC/TG curve (Fig. 1f). To gain an insight into the structure of the carbon layer, Raman spectroscopy was performed (Figure S3). The two strong bands around 1330 and 1600 cm^−1^ could be attributed to the inplane vibrations of disordered amorphous carbon (D band) and crystalline graphic carbon (G band), respectively. The relatively low intensity of D-band to G-band (*I*_D_/*I*_G_ = 0.86) value confirms a certain degree of graphitization of carbon, which is beneficial to the improvement of electrochemical properties for carbon coated composites^33^. Furthermore, the scanning transmission electron microscope-energy dispersive spectrometer (STEM-EDS) elemental mapping (Fig. 2) demonstrates that the Ti, P, O and C atoms are uniformly distributed, which unambiguously reveals the uniformity of carbon coating. That is, a homogeneous and high-quality carbon layer was successfully coated on the surface of LiTi_2_(PO_4_)_3_.

**Figure 3:**
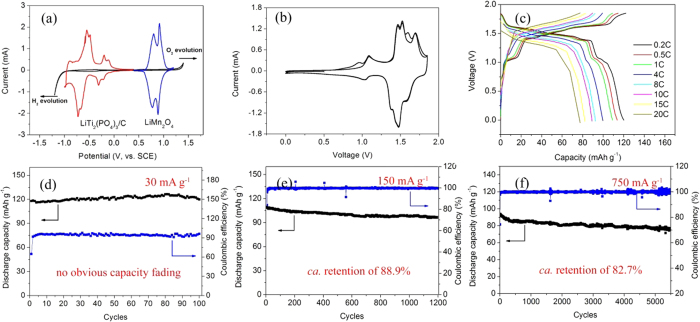
(**a**) Cyclic voltammetry (CV) curves of mesoporous LiTi_2_(PO_4_)_3_@C composite and LiMn_2_O_4_ electrode in Li_2_SO_4_ solution at a sweep rate of 0.4 mV s^−1^, respectively, measured by a three-electrode system using a platinum sheet as the counter electrode and a saturated calomel electrode (SCE) as the reference electrode; (**b**) The first two CV curves of LiTi_2_(PO_4_)_3_@C//LiMn_2_O_4_ ARLB at a sweep rate of 0.4 mV s^−1^; (**c**) Charge and discharge capacities of LiTi_2_(PO_4_)_3_@C//LiMn_2_O_4_ ARLB at various rates (1C = 150 mA g^−1^); Cycling performance and Coulombic efficiency of LiTi_2_(PO_4_)_3_@C//LiMn_2_O_4_ ARLB at 30 mA g^−1^ (**d**), 150 mA g^−1^ (**e**) and 750 mA g^−1^ (**f**), respectively. The capacity was calculated based on the mass of LiTi_2_(PO_4_)_3_ in this paper.

Lithium intercalation and deintercalation behavior of LiTi_2_(PO_4_)_3_@C and LiMn_2_O_4_ electrodes in aqueous electrolyte were investigated by CV measurement (Fig. 3a). The LiTi_2_(PO_4_)_3_@C demonstrates four reduction peaks (*ca.* −0.21 V, −0.31 V, −0.67 V and −0.73 V, respectively) between 0 V and −1.0 V *vs.* SCE. And the corresponding oxidation peaks are located at *ca.* −0.11 V, −0.20 V, −0.48 V and −0.55 V *vs.* SCE, respectively. The excellent kinetics behavior implies the possibility of LiTi_2_(PO_4_)_3_@C as a promising anode for ARLB. Abundant studies have indicated that LiFePO_4_ and LiMn_2_O_4_ could cycle stably in neutral aqueous electrolyte. Accordingly, commercial LiMn_2_O_4_ was directly used in the present work as the cathode in consideration of its relatively high intercalated potential, low cost and excellent cycling stability in aqueous electrolyte. Good lithium insertion/extraction behavior is also observed in Fig. 3a,b depicts the first two CV curves of LiTi_2_(PO_4_)_3_@C//LiMn_2_O_4_ ARLB. In the first cycle, there are six main oxidation peaks (*ca.* 0.95 V, 1.09 V, 1.46 V, 1.52 V, 1.60 V and 1.70 V, respectively) and four reduction peaks (*ca.* 1.03 V, 1.37 V, 1.47 V and 1.58 V, respectively). While the oxidation peaks at 0.95 V and 1.60 V disappear in the second cycle, which may have a relationship with the structure rearrangement of LiTi_2_(PO_4_)_3_@C anode and the details will be discussed in Figure S6. Note that there is no obvious peak corresponding to the evolution of hydrogen or oxygen, consistent with the high Coulombic efficiency (Fig. 3d–f). The rate performance of LiTi_2_(PO_4_)_3_@C is shown in Fig. 3c. It exhibits a discharge capacity of 121 mAh g^−1^ (based on the mass of LiTi_2_(PO_4_)_3_) at 30 mA g^−1^ and 90 mAh g^−1^ at 1500 mA g^−1^, respectively. When the current density is increased to 3000 mA g^−1^, a discharge capacity of 80 mAh g^−1^ is retained with apparent charge/discharge plateaus. The excellent rate capability may originate from the large surface area, abundant mesostructure and high quality carbon coating, which contribute to significantly improved electrode/electrolyte contact area and enhanced conductivity^24^,^34^,^35^. Long-term cycling stability of ARLB at various rates were performed to evaluate the cycling stability of the mesoporous LiTi_2_(PO_4_)_3_@C composite. As seen in Fig. 3f, the LiTi_2_(PO_4_)_3_@C delivers an ultralong cycling life of 5500 cycles with a capacity retention of 82.7% at a current density of 750 mA g^−1^. More importantly, the as-prepared material also shows superior cycling stability at relatively low current densities. At 30 mA g^−1^, the electrode delivers a discharge capacity of 118 mAh g^−1^, and no capacity fading is observed after 100 cycles (Fig. 3d). An initial discharge capacity of 108 mAh g^−1^ and a capacity retention of 88.9% after 1200 cycles are also illustrated at 300 mA g^−1^ in Fig. 3e. The superior cycling stability is further confirmed by the performance at extreme high current density (1500 mA g^−1^, Figure S4). These results demonstrate clearly that LiTi_2_(PO_4_)_3_@C//LiMn_2_O_4_ can be tolerant to various charge/discharge current densities. Poor cycling stability at low current densities is still a critical challenge for ARLB and there are no very clear explanations available so far. It is speculated that the crystal deterioration of electrode, reaction between electrode materials and water or O_2_ and decomposition of water may be the main causes^2^,^12^. The details will be discussed later.

To our best knowledge, the cycling performance of ARLB here has been advanced to a new level, which is much superior to all the reported ARLBs using vanadium oxides, vanadates or LiTi_2_(PO_4_)_3_/C as anode materials to date (see Table S1)^2^,^12^,^14^,^15^. This is a breakthrough for ARLB in term of the cycling life, particularly at a low current density. Note that the ARLB with such superior electrochemical performance can certainly meet the demands of various practical applications. Low Coulombic efficiency due to the decomposition of water and the interaction between aqueous electrolyte and electrode surface^2^,^12^,^20^, is considered as an important origin of capacity fading for ARLB. As demonstrated, the ARLB here can deliver very high Coulombic efficiency at various current densities (~94% at 30 mA g^−1^, >99% at 150 mA g^−1^, 750 mA g^−1^, 1500 mA g^−1^) as shown in Fig. 3(d–f), and Figure S4, in good accordance with the superior cycling performance.

## Discussion

**Figure 4:**
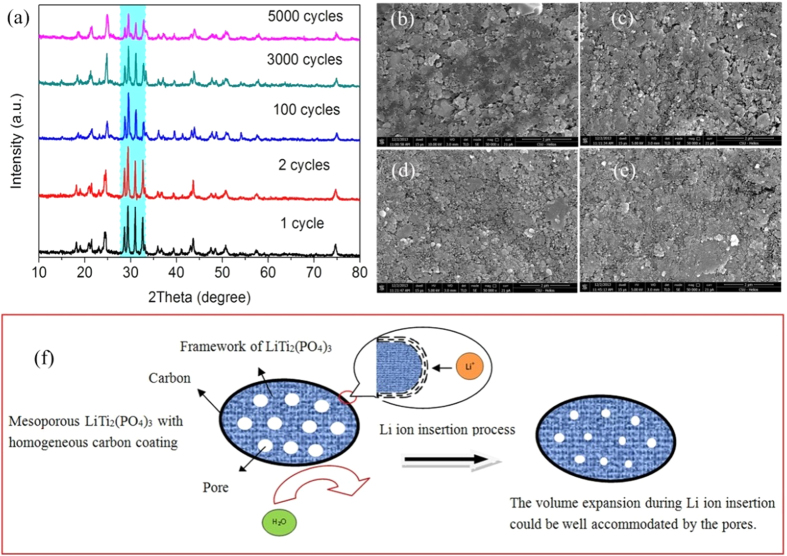
(**a**) XRD patterns of LiTi_2_(PO_4_)_3_/C electrodes after different cycles at 750 mA g^−1^. Before disassembling, each cell was charged to 1.6 V and then kept at that voltage for 2 h; SEM images of LiTi_2_(PO_4_)_3_/C electrode after 5 cycles (**b**), 100 cycles (**c**), 1000 cycles (**d**) and 5000 cycles (**e**) at 750 mA g^−1^; (f) Schematic illustration of the tentative Li ion insertion mechanism in mesoporous LiTi_2_(PO_4_)_3_ with homogeneous carbon coating layer, which can efficiently buffer the volume expansion and avoid H_2_O attacking during repetitive Li^+^ insertion/extraction.

**Figure 5:**
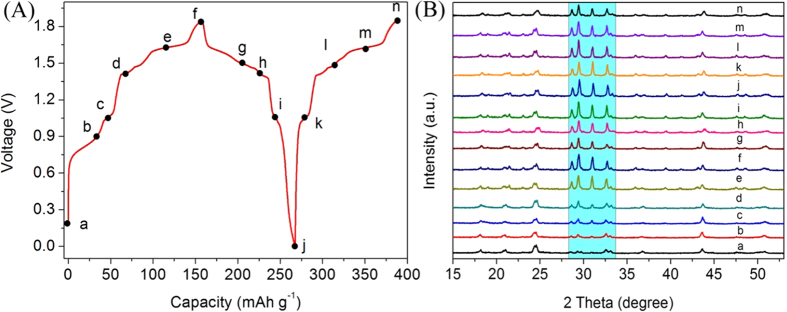
(**A**) The first two charge-discharge curves of LiTi_2_(PO_4_)_3_@C//LiMn_2_O_4_ ARLB at 30 mA g^−1^. (**B**) XRD patterns of LiTi_2_(PO_4_)_3_@C at different cell voltages: a - 0 V, b - 0.9 V, c - 1.0 V, d - 1.4 V, e - 1.6 V, f - 1.85 V, g - 1.5 V, h - 1.4 V, i - 1.05 V, j - 0 V, k - 1.05 V, l - 1.5 V, m - 1.6 V and n - 1.85 V at 30 mA g^−1^.

Structure deterioration, electrode pulverization, and detachment of active material from the conducting environment resulting from the repetitive volume expansion/shrinkage during the Li insertion/extraction are considered as great challenges for long-lifetime battery^36^. For ARLB, H_2_O attacking which leads to the decrease of electrode surface integrity and dissolution of surface active materials is also a fatal cause for capacity fading^28^,^37^. Wang *et al.*^38^ confirmed that the crystalline structure of Li_x_V_2_O_5_ became nearly amorphous after 40 cycles in ARLB. The formation of new compounds was also considered to be the cause for capacity fading of TiP_2_O_7_ by Chen and his co-workers^39^. Caballero *et al.*^37^ considered the dissolution of electrode material as the origin of capacity fading for ARLB. Therefore, the structures of LiMn_2_O_4_ cathodes (Figure S5) and LiTi_2_(PO_4_)_3_@C anodes (Fig. 4a) after different cycles (1, 2, 100, 3000 and 5000) were examined by XRD. Same as reported in the references, the LiMn_2_O_4_ cathodes used here show good structure stability in ARLB (Figure S5). To find out the reasons for such good structure stability, the structure and surface morphology evolution of LiTi_2_(PO_4_)_3_@C anodes were investigated in details. As the cycling proceeds, the intensities of diffraction peaks located at 2θ = 28.7°, 29.5°, 31.0° and 32.6° decrease gradually. However, there are no new impurity peaks for the electrodes after different cycles in comparison with that after 1 cycle, implying excellent structure stability of LiTi_2_(PO_4_)_3_@C anode. Note that there is a slight difference for the XRD patterns of cycled LiTi_2_(PO_4_)_3_@C electrode in comparison with the LiTi_2_(PO_4_)_3_@C powder^40^. With this regard, XRD patterns of LiTi_2_(PO_4_)_3_@C electrodes at different states (marked as a-n) in the first two cycles are given in Fig. 5. It should be noted that the diffraction lines at 28.7°, 29.5°, 31.0° and 32.6° in mark a are much weaker than that at 24.4°. During the first charge process (a–f), the intensities of these lines increase obviously with the rising of cell voltage and gradually become the main ones. In the following process (g–n), their diffraction intensities change slightly. Compared with those in the first charge process, the intensities of the diffraction lines at 28.7°, 29.5°, 31.0° and 32.6° in the second charge process are different, implying a structure rearrangement of LiTi_2_(PO_4_)_3_@C after the first cycle. Structure information is consistent with the first two CV curves in Fig. 3b. The CV curves (Figure S6) of LiTi_2_(PO_4_)_3_@C//LiMn_2_O_4_ after 5000 cycles further confirms good Li ion insertion/extraction kinetics after long-term cycling. The surface microstructures of LiTi_2_(PO_4_)_3_@C electrodes after 5, 100, 1000 and 5000 cycles at 750 mA g^−1^ are compared in Fig. 4. The LiTi_2_(PO_4_)_3_@C electrode surface after 5000 cycles still remains intact in comparison with that after 5 cycles, implying the negligible effect of H_2_O attacking. That is, a relatively stable electrode surface and effective suppression of active materials dissolution have been achieved for the as-prepared LiTi_2_(PO_4_)_3_@C. Figure S7 demonstrates a slight increase of R_ct_ after 5000 cycles, which is in agreement with the capacity fading for ARLB. Generally, the possible capacity fading mechanisms of bare LiTi_2_(PO_4_)_3_ and LiTi_2_(PO_4_)_3_ with heterogeneous carbon coating are illustrated in Figure S8, demonstrating the inferior cycling stability during the cycling process. Hence, according to the discussions above, a tentative Li ion insertion mechanism in mesoporous LiTi_2_(PO_4_)_3_ with homogeneous carbon coating layer is proposed in Fig. 4f. That is, the mesoporous structure confirmed to be more stable than the more common ones (*e.g.,* bulk, nanoparticle)^23^,^35^,^41^, could provide enough void space to accommodate the volume expansion during cycling and the outer high-quality carbon coating layer could withstand the attacking of H_2_O, thus resulting in stable crystal structure and electrode surface during the long term cycling (Fig. 4f).

In summary, we have developed a solvothermal method accompanied with an advanced carbon coating strategy to synthesize mesoporous LiTi_2_(PO_4_)_3_@C composite. When used as an anode for ARLB, the electrode delivered an ultra-long cycling life up to 5500 cycles at 750 mA g^−1^. Even at a relatively low current density of 30 mA g^−1^, no obvious capacity fading was observed after 100 cycles. This is a breakthrough in the cycling stability of ARLB at both high and low current densities, which should be mainly ascribed to the high performance LiTi_2_(PO_4_)_3_@C anode. The mesoporous structure, large surface area, high-quality carbon coating layer and the stable 3D crystal structure have been verified as important factors. By virtues of its superior electrochemical performance, the mesoporous LiTi_2_(PO_4_)@C composite prepared in the present study could be considered as a very promising candidate as an anode for ARLB.

## Methods

### Synthesis of LiTi_2_(PO_4_)_3_@C composite

All the starting materials were analytically pure grade and directly used without any purification. A novel two-step strategy involving a solvothermal method and a following carbon coating process was employed. Certain amounts of lithium hydroxide, titanium sulfate and ammonia phosphate (NH_4_H_2_PO_4_) with the molar ratio of 2.7: 2: 4.5 were dissolved in ethylene glycol in advance, respectively. The lithium hydroxide and ammonia phosphate solution were first mixed quickly and stirred for 3 h. The titanium sulfate ethylene glycol solution was then gradually added into the mixed solution. After stirring for 0.5 h, the suspension was transferred into a 100 ml Teflon lined stainless steel autoclave. The autoclave was sealed and heated at 160 °C for 10 h and then cooled to room temperature naturally. The white precipitates were collected by centrifugation, and washed with distilled water several times and then dried at 80 °C overnight. Following that, 0.3 g of the white precursor was dispersed well in distilled water in the presence of sodium dodecylsulfonate (10 g/L) and 0.2 g of phenolic resin powder was dissolved in absolute ethanol. The phenolic resin solution was dropwise added into the suspension of LiTi_2_(PO_4_)_3_ precursor. The mixed suspension was heated to 50 °C on a hotplate with stirring till the ethanol evaporated. In this way, the phenolic resin could be covered well on the surface of LiTi_2_(PO_4_)_3_ precursor. After the centrifugation and washing several times by distilled water, the obtained solid was dried at 80 °C overnight and then calcined at 700 °C for 5 h with a ramping rate of 5 °C/min in a mixed flow of H_2_/Ar (5:95, v/v).

### Characterizations

All X-ray diffraction (XRD) data were examined by the X-ray diffractometer (Dandong Haoyuan, DX-2700) utilizing a Cu-Kα1 source with a step of 0.02°. XRD measurement of electrodes was different from the examination of the powder. After being washed with distilled water and dried for several hours, the whole electrode consisting of active material, Super P carbon and polytetrafluoroethylene (PTFE) was directly used to perform the XRD measurements and it is worthy to note that no signal of stainless steel mesh was observed probably due to the thick electrode film. Each cell was charged to 1.6 V and kept at that voltage for 2 h before disassembling. The XPS patterns were collected using Al Kα radiation at a voltage of 12 kV and current of 6 mA. Charging effect was corrected by adjusting the binding energy of C1s peak from carbon contamination to 284.5 eV. Microstructural studies of electrodes after different cycles were conducted using a Nova NanoSEM 230 SEM. TEM, high resolution TEM (HRTEM) images and STEM-EDS elemental mapping of as-prepared LiTi_2_(PO_4_)_3_@C powders were obtained using a FEI Tecnai G2 F20 S-TWIX TEM. The BET surface area of the samples was detected by nitrogen adsorption/desorption at −196 °C using a Builder SSA-4200 apparatus. The pore size distributions for LiTi_2_(PO_4_)_3_@C were obtained by the Barrett-Joyner-Halenda (BJH) method. The XPS fitting was performed using XPSPEAK software, and the crystal structure of LiTi_2_(PO_4_)_3_ was drawn by Diamond 3.2.

### Electrochemical measurements

The used LiMn_2_O_4_ was provided by Hunan Reshine New Material Co., Ltd. The LiTi_2_(PO_4_)_3_@C and LiMn_2_O_4_ electrodes were made in a similar way. Tested electrodes were obtained by pressing a mixture of the active material, Super P carbon and PTFE in a weight ratio of 80:10:10 using distilled water as solvent on a stainless steel mesh and then dried at 110 °C for 8 h. Cyclic voltammetry (CV) of LiTi_2_(PO_4_)_3_@C anode and LiMn_2_O_4_ cathode was performed using a three electrode system, respectively, where the tested electrode was used as working electrode, platinum sheet electrode as the counter electrode and saturated calomel electrode (SCE, 0.242 V *vs.* SHE: standard hydrogen electrode) as reference electrode. CV test was investigated at room temperature using an electrochemical station (CHI660D). The CR2016 coin-type cells were constructed by using LiMn_2_O_4_ electrode as cathode, LiTi_2_(PO_4_)_3_@C electrode as anode, 2 mol L^−1^ Li_2_SO_4_ as electrolyte. Excessive LiMn_2_O_4_, with cathode/anode mass ratio of (1.5~2.0)/1 was designed for exactly evaluating the electrochemical properties of LiTi_2_(PO_4_)_3_@C. The Li_2_SO_4_ electrolyte was pre-treated by the flowing argon injection into the solution to eliminate the soluble oxygen. Charge and discharge tests were conducted under a desired current density by a Neware battery testing system (CT-3008W) at room temperature. Electrochemical impedance spectroscopy (EIS) was recorded by a Princeton workstation (PARSTAT2273, EG&G, US) over the frequency range from 100 kHz to 10 mHz with an amplitude of 5 mV. Before testing, the measured cell was charged to 1.6 V at 30 mA g^−1^, and then kept for a period of time to reach a stable state.

## Additional Information

**How to cite this article**: Sun, D. *et al.* Long-lived Aqueous Rechargeable Lithium Batteries Using Mesoporous LiTi_2_(PO_4_)_3_@C Anode. *Sci. Rep.* **5**, 17452; doi: 10.1038/srep17452 (2015).

## Supplementary Material

Supplementary Information
