# Supplementary material for: Long-lived Aqueous Rechargeable Lithium Batteries Using Mesoporous LiTi2(PO4)3@C Anode
Source: Sci Rep. 2015 Dec 9;5:17452. doi: 10.1038/srep17452 (PMC4673533; doi:10.1038/srep17452)
Supplement: Supplementary Information [file srep17452-s1.doc]

Supporting Information

# Long-lived Aqueous Rechargeable Lithium Batteries Using Mesoporous LiTi_2_(PO_4_)_3_@C Anode

Dan Sun^1^, Yougen Tang^1,2^, Kejian He^2^, Yu Ren^3^, Suqin Liu^1^ & Haiyan Wang^1,2,4*^

^1^ College of Chemistry and Chemical Engineering, Central South University, Changsha, 410083, P.R. China.

^2^ Advanced Research Centre Central South University, Changsha, 410083, P.R. China.

^3^ Battery Materials, Basf China Limited, Shanghai, 201206, P.R. China

^4^ State Key Laboratory for Powder Metallurgy, Central South University, Changsha 410083, P.R. China


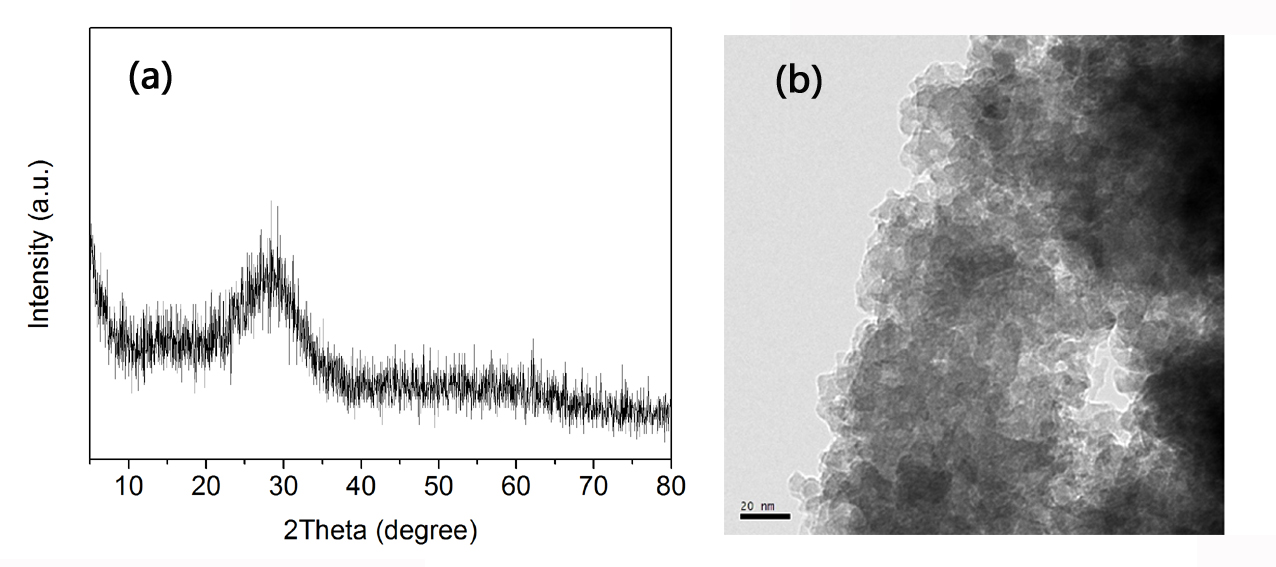


Figure S1 XRD pattern (a) and TEM image (b) of as-prepared precursor

Figure S2 High resolution XPS spectrum of Ti in as-prepared LiTi_2_(PO_4_)_3_@C

Figure S3 Raman spectrum of as-prepared LiTi_2_(PO_4_)_3_@C

Figure S4 Cycling performance and Coulombic efficiency of LiTi_2_(PO_4_)_3_@C//LiMn_2_O_4_ at 1500 mA g^-1^

Figure S5 XRD of LiMn_2_O_4_ electrodes after different cycles

Figure S6 Cyclic voltammetry curves of LiTi_2_(PO_4_)_3_@C//LiMn_2_O_4_ at 0.4 mV s^-1^, which has been tested for 5000 cycles at 750 mA g^-1^.

Figure S7 The Nyquist plots of the LiTi_2_(PO_4_)_3_@C//LiMn_2_O_4_ ARLB after different cycles at 750 mA g^-1^.

As displayed, the plots consist of a depressed semicircle in the high frequency regions relating to the charge-transfer impedance (R*_ct_*) on electrode-electrolyte interface and a straight line in the low frequency region which is ascribed to the Warburg impedance. The R*_ct_* impedance of cell after 5000 cycles is slightly larger compared with that of the cell after 10 cycles, which corresponds well with the capacity fading. Increase of R*_ct_* was considered as the important factor for the capacity fading of cathode materials*(2)*. As is well known, R_ct_ has correlation with many factors such as electronic conductivity, crystal structure, the inter-particle contacts and electrode surface condition[^3^](#_ENREF_3).


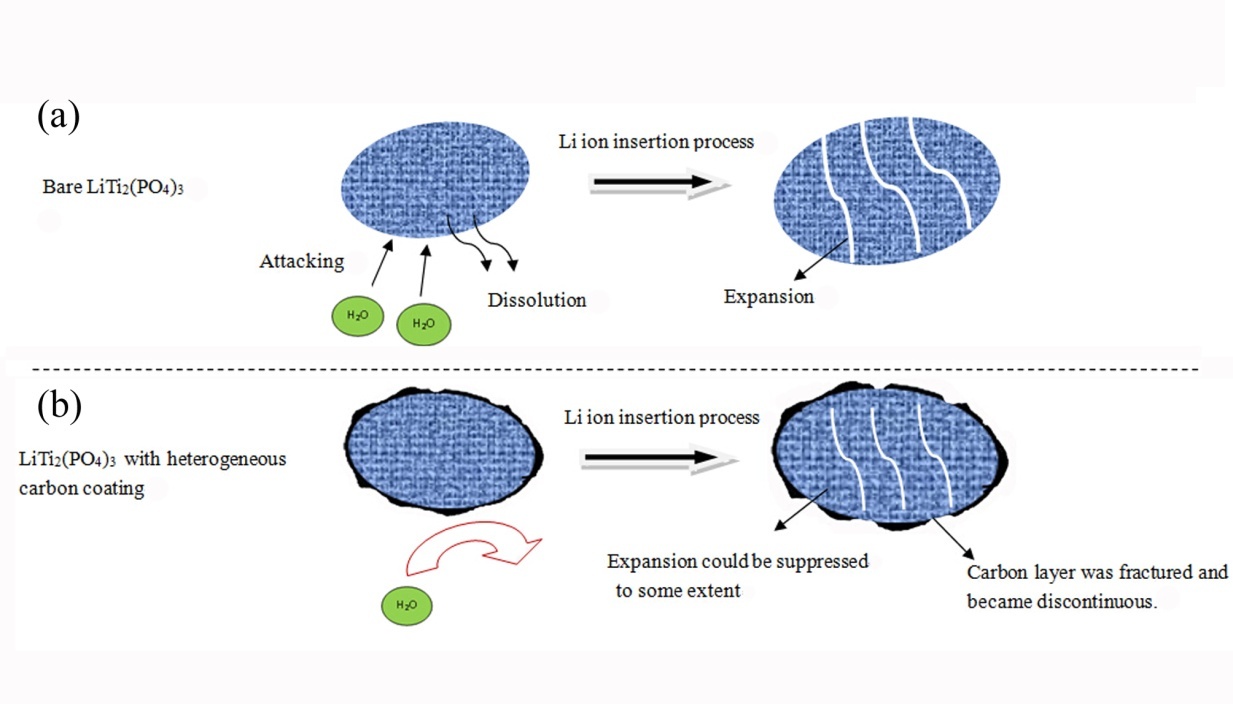


Figure S8 The possible fading mechanism of bare LiTi_2_(PO_4_)_3_ (a) and LiTi_2_(PO_4_)_3_ with heterogeneous carbon coating (b).

As seen, the bare LiTi_2_(PO_4_)_3_ electrode always suffers from the attacking of H_2_O resulting the dissolution of active materials. Moreover, during the Li ion insertion process, the crystal cell expands, which will cause the deterioration of crystal structure. Hence, the capacity of electrode fades seriously. Although a heterogeneous carbon coating layer could protect the electrode from the attacking of H_2_O and suppress the expansion of crystal structure to some extent, the thin parts of the carbon layer are easy to be fractured and become discontinuity. Thus the heterogeneous carbon coating is not sufficient to enhance the stability of electrode.

Table S1 Cycling performance of ARLB under different test conditions reported by different research groups

| No. | Battery system | Capacity retention/Cycles | Rate | Ref. |
| --- | --- | --- | --- | --- |
| 1 | VO_2_(B)/C//LiMn_2_O_4_ | Failed/25 | 0.69 mA cm^-2^ | [^4^](#_ENREF_4) |
| 2 | V_2_O_5_·nH_2_O//LiMn_2_O_4_ | 89%/100 | 50 mA g^-1^ | [^5^](#_ENREF_5) |
| 3 | H_2_V_3_O_8_^*^ | 72%/50 | 0.1 A g^−1^ | [^6^](#_ENREF_6) |
| 4 | LiV_3_O_8_//LiMn_2_O_4_ | 78.7%/50 | 0.2 C | [^7^](#_ENREF_7) |
| 5 | Polyaniline//LiMn_2_O_4_ | 81.4%/150 | 75 mA g^-1^ | [^8^](#_ENREF_8) |
| 6 | LiV_3_O_8_//LiCo_2_O_4_ | 36%/100 | 3.4 mA cm^-2^ | [^9^](#_ENREF_9) |
| 7 | LiV_3_O_8_//LiNi_0.81_Co_0.19_O_2_ | 40%/100 | 1 mA cm^-2^ | [^10^](#_ENREF_10) |
| 8 | TiP_2_O_7_//LiCo_2_O_4_ | 37%/25 | 0.1C | [^11^](#_ENREF_11) |
| 9 | LiV_3_O_8_^*^ | 30.85%/100 | 0.1C | [^12^](#_ENREF_12) |
| 10 | LiV_3_O_8_//LiMn_2_O_4_ | 53.5%/100 | 0.2C | [^13^](#_ENREF_13) |
| 11 | Li_1.2_V_3_O_8_^*^ | 88%/50 | 0.2C | [^14^](#_ENREF_14) |
| 12 | Polypyrrole-coated LiV_3_O_8_ | 84%/10 | 250 mA cm^-2^ | [^1^](#_ENREF_1) |
| 13 | Na_2_V_6_O_16_·0.14H_2_O//LiMn_2_O_4_ | 77%/200 | 300 mA g^-1^ | [^15^](#_ENREF_15) |
| 14 | NaV_6_O_15_//LiMn_2_O_4_ | 80%/400 | 300 mA g^-1^ | [^16^](#_ENREF_16) |
| 15 | LiTi_2_(PO_4_)_3_/C//LiMn_2_O_4_ | 82%/200 | 10 mA cm^-2^ | [^17^](#_ENREF_17) |
| 16 | LiTi_2_(PO_4_)_3_/C//LiFePO_4_ | 90%/1000 | 6 C | [^18^](#_ENREF_18) |
|  |  | 85%/50 | ~0.125C |  |
| 17 | LiTi_2_(PO_4_)_3_/C//LiMn_2_O_4_ | 75%/10 | 0.1C | [^11^](#_ENREF_11) |
| 18 | LiTi_2_(PO_4_)_3_//LiMn_0.05_Ni_0.05_Fe_0.9_PO_4_ | <80%/50 | 0.2 mA cm^-2^ | [^19^](#_ENREF_19) |
| 19 | LiTi_2_(PO_4_)_3_/C^*^ | 90%/100 | 0.2C | [^20^](#_ENREF_20) |
| 20 | LiTi_2_(PO_4_)_3_//LiNi_1/3_Mn_1/3_Co_1/3_O_2_ | 92%/200 | 0.2 mA cm^-2^ | [^21^](#_ENREF_21) |
| 21 | MoO_3_/PPy// LiMn_2_O_4_ | 90%/150 | 1000 mA g^-1^ | [^22^](#_ENREF_22) |
| 22 | LiTi_2_(PO_4_)_3_@C//LiMn_2_O_4_ | 90%/300 | 0.2C | [^23^](#_ENREF_23) |
|  |  | 84%/1300 | 1C |  |
| 23 | LiTi_2_(PO_4_)_3_@C//LiMn_2_O_4_ | ~100%/100  88.9%/1200 | 30 mA g^-1^ (0.2C)  150 mA g^-1^(1C) | this work |
|  |  | 82.7%/5500 | 750 mA g^-1^ (5C) |  |

* The electrochemical properties of materials are tested using a three-electrode system.

**References**

1. Liu L. L., Wang X. J., Zhu Y. S., Hu C. L., Wu Y. P., Holze R. Polypyrrole-coated LiV_3_O_8_-nanocomposites with good electrochemical performance as anode material for aqueous rechargeable lithium batteries. *J. Power Sources* **224**, 290-294 (2013).

2. Nobili F, Croce F, Scrosati B, Marassi R. Electronic and Electrochemical Properties of Li_x_Ni_1-y_Co_y_ O_2_ Cathodes Studied by Impedance Spectroscopy. *Chem. Mater.* **13**, 1642-1646 (2001).

3. Fan J, Fedkiw PS. Electrochemical impedance spectra of full cells: Relation to capacity and capacity-rate of rechargeable Li cells using LiCoO_2_, LiMn_2_O_4_, and LiNiO_2_ cathodes. *Journal of Power Sources* **72**, 165-173 (1998).

4. Li W., Dahn J. R., Wainwright D. S. Rechargeable lithium batteries with aqueous electrolytes. *Science* **264**, 1115-1118 (1994).

5. Stojković I., Cvjetićanin N., Pašti I., Mitrić M., Mentus S. Electrochemical behaviour of V_2_O_5_ xerogel in aqueous LiNO_3_ solution. *Electrochem. Commun.* **11**, 1512-1514 (2009).

6. Li H., Zhai T., He P., Wang Y., Hosono E., Zhou H. Single-crystal H_2_V_3_O_8_ nanowires: a competitive anode with large capacity for aqueous lithium-ion batteries. *J. Mater. Chem.* **21**, 1780-1787 (2011).

7. Zhao M., Zheng Q., Wang F., Dai W., Song X. Electrochemical performance of high specific capacity of lithium-ion cell LiV_3_O_8_//LiMn_2_O_4_ with LiNO_3_ aqueous solution electrolyte. *Electrochim. Acta* **56**, 3781-3784 (2011).

8. Liu L., Tian F., Zhou M., Guo H., Wang X. Aqueous rechargeable lithium battery based on polyaniline and LiMn_2_O_4_ with good cycling performance. *Electrochim. Acta* **70**, 360-364 (2012).

9. Wang G., Fu L., Zhao N., Yang L., Wu Y., Wu H. An aqueous rechargeable lithium battery with good cycling performance. *Angew. Chem. Int. Ed.* **46**, 295-297 (2007).

10. Köhler J., Makihara H., Uegaito H., Inoue H., Toki M. LiV_3_O_8_: characterization as anode material for an aqueous rechargeable Li-ion battery system. *Electrochim. Acta* **46**, 59-65 (2000).

11. Wang H., Huang K., Zeng Y., Yang S., Chen L.. Electrochemical properties of TiP_2_O_7_ and LiTi_2_(PO_4_)_3_ as anode material for lithium ion battery with aqueous solution electrolyte. *Electrochim. Acta* **52**, 3280-3285 (2007).

12. Liu L., Tian F., Yang Z., Wang X., Zhou M., Wang X. Electrochemical behavior of nanostructured LiV_3_O_8_ in aqueous LiNO_3_ solution. *J. Phys.Chem. Solids* **72**, 1495-1500 (2011).

13. Wang G. J., Zhang H. P., Fu L. J., Wang B., Wu Y. P. Aqueous rechargeable lithium battery (ARLB) based on LiV_3_O_8_ and LiMn_2_O_4_ with good cycling performance. *Electrochem. Commun.* **9**, 1873-1876 (2007).

14. Stojkovic I., Cvjeticanin N., Mitric M., Mentus S. Electrochemical properties of nanostructured Li_1.2_V_3_O_8_ in aqueous LiNO_3_ solution. *Electrochim. Acta* **56**, 6469-6473 (2011).

15. Zhou D., Liu S., Wang H., Yan G. Na_2_V_6_O_16_·0.14H_2_O nanowires as a novel anode material for aqueous rechargeable lithium battery with good cycling performance. *J. Power Sources* **227**, 111-117 (2013).

16. Sun D*, et al.* Aqueous rechargeable lithium batteries using NaV_6_O_15_ nanoflakes as high performance anodes. *J. Mater. Chem. A* **2**, 12999-13005 (2014).

17. Luo J. Y., Xia Y. Y. Aqueous lithium-ion battery LiTi_2_(PO_4_)_3_/LiMn_2_O_4_ with high power and energy densities as well as superior cycling stability. *Adv. Funct. Mater.* **17**, 3877-3884 (2007).

18. Luo J. Y., Cui W. J., He P., Xia Y. Y. Raising the cycling stability of aqueous lithium-ion batteries by eliminating oxygen in the electrolyte. *Nat.Chem.* **2**, 760-765 (2010).

19. Liu X. H., Saito T., Doi T., Okada S., Yamaki J. I. Electrochemical properties of rechargeable aqueous lithium ion batteries with an olivine-type cathode and a Nasicon-type anode. *J. Power Sources* **189**, 706-710 (2009).

20. Wessells C., La Mantia F., Deshazer H., Huggins R. A., Cui Y. Synthesis and electrochemical performance of a lithium titanium phosphate anode for aqueous lithium-ion batteries. *J. Electrochem. Soc.* **158**, A352-A355 (2011).

21. Shivashankaraiah R. B., Manjunatha H., Mahesh K. C., Suresh G. S., Venkatesha T. V. Electrochemical characterization of LiTi_2_(PO_4_)_3_ as anode material for aqueous rechargeable lithium batteries. *J. Electrochem. Soc.* **159**, A1074-A1082 (2012).

22. Tang W., Liu L., Zhu Y., Sun H., Wu Y., Zhu K. An aqueous rechargeable lithium battery of excellent rate capability based on a nanocomposite of MoO_3_ coated with PPy and LiMn_2_O_4_. *Energy Environ. Sci.* **5**, 6909-6913 (2012).

23. Sun D*, et al.* Advanced aqueous rechargeable lithium battery using nanoparticulate LiTi_2_(PO_4_)_3_/C as a superior anode. *Sci Rep* **5**, (2015).
